# From a retrovirus infection of mice to a long noncoding RNA that induces proto-oncogene transcription and oncogenesis via an epigenetic transcription switch

**DOI:** 10.1038/sigtrans.2016.7

**Published:** 2016-05-13

**Authors:** Alan Garen

**Affiliations:** 1 Department of Molecular Biophysics and Biochemistry, Yale University, New Haven, CT, USA; 2 Sichuan University, Department of Obstetric and Gynecologic, West China Second University Hospital, Chengdu, China; 3 State Key Laboratory of Biotherapy, West China Hospital, Chengdu, China

## Abstract

Here I review the properties of the mouse retroelement VL30-1, which apparently derived from retrotranspostions of a founder VL30 retrovirus that infected the mouse germline after the mouse–human speciation. The *VL30-1* gene is transcribed as a long noncoding RNA (lncRNA) with an essential host function in an epigenetic transcription switch (ETS) that regulates transcription of multiple genes, including proto-oncogenes that control cell proliferation and oncogenesis. The ETS involves the tumor suppressor protein PSF that has a DNA-binding domain (DBD) and two RNA-binding domains (RBDs). The DBD binds to promoters that have a DBD-binding sequence and switches off transcription, and the RBDs bind lncRNAs that have a RBD-binding sequence, releasing PSF and switching on transcription. VL30-1 lncRNA has two RBD-binding sequences, apparently acquired by mutations during retrotranspositions of the founder retrovirus, which drive proto-oncogene transcription and oncogenesis via the ETS. VL30-1 lncRNA is a seminal example of the key role of endogenous retroviruses (ERVs) and their retroelements in the evolution of transcription regulatory systems.

The operon model of gene regulation, a founding concept of molecular biology proposed by Jacob and Monod in 1961 based on their studies with *Escherichia coli*,^1^ focused attention on protein-coding genes as the fundamental functional component of all genomes, as affirmed in Monod’s statement that ‘anything found to be true for *E. coli* must also be true for the elephant.’ Although it was known that mammalian genomes also contained DNA that did not encode any proteins, such DNA was usually called useless or selfish.^2,3^ The revelation from whole-genome sequencing that protein-coding genes comprise only a minuscule part of a mammalian genome, ~2% of the human and mouse genomes,^4–6^ was a wake-up call to understand how most of the genomic DNA survived evolutionary selection for the fittest organisms. An associated revelation was that ~8–10% of the human and mouse genomes consist of ERVs presumably derived from retroviral infections of the mammalian germlines.^7–9^ An ERV initially functions as a selfish DNA that integrates at multiple genomic sites via successive cycles of duplicative retrotansposition (DRT), involving integration, transcription, reverse transcription and integration at another genomic site, which must eventually be suppressed in order for the host to survive, while the ERV must acquire a beneficial host function to survive as a component of the host genome.

Here I discuss the remarkable properties of a mouse ERV called VL30-1,^10^ a member of the VL30 ERV family^11^ that probably originated from an infection of the mouse germline by a founder retrovirus after mouse–human speciation, as there are no VL30-related sequences in the human genome. The mouse genome currently is estimated to contain 150–200 VL30-related sequences, ranging from a full-length 5–6-kbp *VL30* gene that has the features of an ERV, notably 5′ and 3′ long terminal repeats (LTRs), to a single ‘solo’ LTR.^11,12^ In the full-length VL30 genes sequenced so far, including VL30-1, the internal DNA flanked by the LTRs contains multiple mutations, including stop codons in all three reading frames, which block translation of the retroviral proteins required for further DRT cycles. Although the DRT cycles are suppressed, at least some of the full-length VL30 genes, including VL30-1, are transcribed as a lncRNA with a poly-A tail and are exported to the cytoplasm.

The VL30-1 lncRNA was discovered in an experiment involving transfection of a human tumor cell by a retroviral vector produced in a mouse cell containing VL30-1 lncRNA, resulting in encapsulation of VL30-1 lncRNA in the retroviral particles and integration in the host genome as an ERV, which increased the metastatic potential of the host.^10^ Further studies showed that the increase in metastatic potential was caused by a novel mechanism of gene regulation involving the protein PSF^13^ and a PSF-binding RNA.^14–17^ PSF contains a DBD and two homologous RBDs (RBD-1 and RBD-2; Figure 1). The DBD in PSF binds to the promoter of a gene containing a DBD-binding site and represses transcription,^14,18^ and the RBD-1 and RBD-2 bind RNAs, usually a lncRNA, and reverse repression by PSF (Figure 2).^14–17^ This mechanism of gene regulation, which I term an ETS, regulates the transcription of multiple genes, including proto-oncogenes that control cell division and proliferation, and the *P450scc* gene that controls steroid synthesis.^14–17^ The *PSF* gene is strongly conserved between mice and humans,^19^ whereas the major PSF-binding RNAs differ yet retain the same function in the ETS. The major PSF-binding RNA in mice is VL30-1 lncRNA, which has the features of a LTR retroelement (Figure 3). VL30-1 lncRNA has an essential function in driving cell proliferation during mouse development, and it also has a deleterious function in inducing the oncogenic transformation of normal cells.^14–17^ The PSF-binding sequences in VL30-1 lncRNA are localized in two short homologous regions, one with 22 nucleotides and another with 30 nucleotides (Figure 4), which probably were generated during the DRT cycles. Because VL30-1 lncRNA is exported to the cytoplasm after transcription (unpublished data), it must be imported back to the nucleus to bind to PSF; the import mechanism is not known.

The *PSF* gene probably existed in the mouse genome before the retrovirus infection that generated the *VL30-1* gene. Although PSF protein is expressed during early development when cells proliferate, it does not function as a repressor until cells begin to differentiate and proliferation stops (unpublished data). Consequently, another PSF-binding lncRNA RNA, probably MALAT-1,^20,21^ was needed before VL30-1 lncRNA was available, to prevent binding of PSF to proto-oncogenes during early development. As VL30-1 lncRNA binds more effectively to PSF than MALAT-1 lncRNA (unpublished data), it could have co-opted the role of MALAT-1 lncRNA as the major PSF-binding RNA in the mouse ETS, providing an explanation for the surprising finding that MALAT-1 lncRNA is dispensable for mouse development and survival.^22,23^ I propose that the beneficial function of VL30-1 lncRNA, which was needed for its evolutionary survival in the mouse genome, was achieved in this way, providing a seminal example of the importance of retroviruses and their retroelement descendants in shaping the evolution of epigenetic systems for regulating gene transcription.

## Figures and Tables

**Figure 1:**
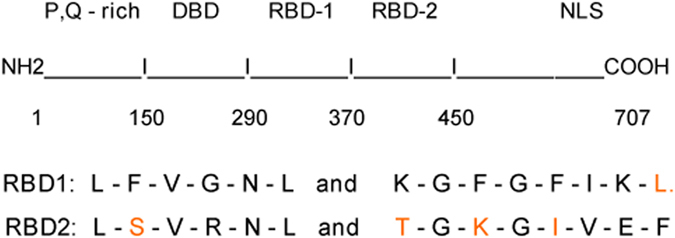
Organization of the PSF protein. The upper panel shows the DNA-binding domain (DBD) followed by the two RNA-binding domains (RBD-1 and RBD-2) and nuclear-localization signals (NLSs).^13,19,24^ The lower panel shows the RBD consensus residues in black and the non-consensus residues in red.^19,25^

**Figure 2:**
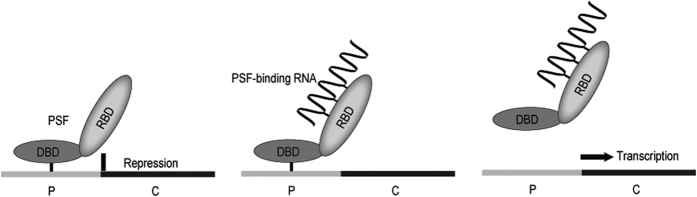
Regulation of transcription by PSF protein and PSF-binding RNA. The first diagram on the left shows the binding of the DBD in PSF to the promoter (P) of a gene, causing repression of transcription of the coding region (C). The second diagram in the center shows the binding of a RNA molecule to the RBD-1 and RBD-2 regions in PSF. The third diagram on the right shows the release of PSF from the promoter and initiation of transcription.^16^

**Figure 3:**
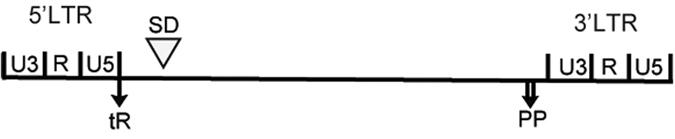
Map of VL30-1 lncRNA. The map shows the 5′ and 3′ LTRs, tR (tRNA primer binding site), SD (splice donor site) and PP (polypurine tract).^10^

**Figure 4:**
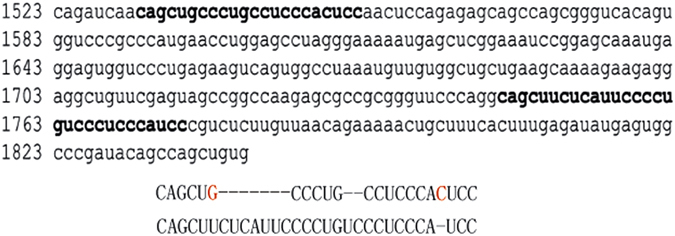
PSF-binding sequences in VL30-1 lncRNA.^14^ The upper panel shows the two PSF-binding sequences (in bold) located in the region spanning nucleotides 1523–1841 of the full-length VL30-1 lncRNA, which contains 4939 nucleotides. The lower panel shows a comparison of the identical nucleotides in the two PSF-binding sequences (in black) and the non-identical nucleotides (in red).
